# Effect of dietary selenium source (organic vs. mineral) and muscle pH on meat quality characteristics of pigs

**DOI:** 10.1002/fsn3.368

**Published:** 2016-06-01

**Authors:** Luis Calvo, Fidel Toldrá, Ana I. Rodríguez, Clemente López‐Bote, Ana I. Rey

**Affiliations:** ^1^ IncarlopsaCtra. N‐400 km. 95400Tarancón, Cuenca16400Spain; ^2^ Instituto de Agroquímica y Tecnología de Alimentos (CSIC)Av Agustín Escardino 7Paterna (Valencia)46980Spain; ^3^ Dpto. Producción AnimalFacultad de Veterinaria, Universidad Complutense de MadridMadrid28040Spain

**Keywords:** **C**olor stability, drip loss, organic selenium, pH, TBARS

## Abstract

This study evaluates the effect of organic (Se‐enriched yeast; SeY) versus inorganic selenium (sodium selenite; SeS) supplementation and the different response of selenium source according to muscle pH on pork meat quality characteristics. Pigs (*n* = 30) were fed the Se‐supplemented diets (0.3 mg/kg) for 65 days. Neither electric conductivity (EC) nor drip loss were affected by the selenium source. The SeY group had lower TBARS in muscle samples after day 7 of refrigerated storage and higher a* values on days 1 and 7 than the SeS group. The effect of dietary selenium source on some meat quality characteristics was affected by muscle pH. Hence, as the muscle pH increases, the drip loss decreases but this effect is more marked with the dietary organic Se enrichment. Muscle pH seems to modulate the action of selenium in pork, especially some meat characteristics such as drip loss.

## Introduction

Selenium (Se) has become an interesting nutrient in animal production because it improves the nutritional value and quality characteristics of meat products (Surai 2006). This trace mineral was identified as a component of the antioxidant enzyme glutathione peroxidase (GSH‐Px) (Rotruck et al. 1973), which participates in redox regulation by removing and decomposing hydrogen peroxide and lipid hydroperoxides using glutathione as electron donor (Hayes and McLellan 1999). Consequently, selenium deficiency has been associated with diseases induced by increased oxidative stress such as various types of muscular dystrophy (Rederstorff et al. 2006), and in human increased susceptibility to some degenerative diseases such as cancer (Gramadzinska et al. 2008). These antioxidant functions of Se have also been shown to persist in postmortem muscle tissue (Mahan et al. 2014). Hence, various dietary strategies in animal feeding have been developed for providing Se‐enriched meat in order to increase human selenium intake (Zhang et al. 2010).

Se is commonly added to pig diets as sodium selenite (SeS; Na_2_SeO3), an inorganic form. However, there has been increasing interest in organic Se in recent years because of its higher absorption and biological effectiveness in pigs (Mahan et al. 1999, 2014; Jang et al. 2010), broilers (Mikulski et al. 2009; Briens et al. 2013), turkeys (Juniper et al. 2011), cows (Juniper et al. 2008), and more recently laying hens (Delezie et al. 2014). Organic Se has also been reported to have higher antioxidant activity, whereas the inorganic form may act as a prooxidant (Spallholz 1994) and have toxic effects particularly at high levels (Seko et al. 1989). Antioxidant functions of organic Se are also more effective in delaying postmortem oxidation reactions (Mahan et al. 2014), which affects adversely the nutritional value, flavor, and shelf life of meat products (Morrissey et al. 1998). In addition, other meat quality characteristics such as water‐holding capacity or color, which are considered useful parameters for evaluating quality and consumer acceptability, were affected by organic Se supplementation (Mahan et al. 1999; Zhan et al. 2007). However, the organic form (from Se‐enriched yeast) used in the feed industry does not always show positive effects on meat quality characteristics (Juniper et al. 2008, 2011; Kawecka et al. 2013; Lisiak et al. 2014). Organic forms such as Se‐enriched yeast (SeY), are taken up via methionine transporter mechanisms and can be incorporated to selenoenzymes or in place of methionine into general body proteins (Suzuki and Ogra 2002) more readily than the inorganic form (Surai 2006). Se utilization and its effects on meat quality may also be affected by muscle characteristics since PSE filets from chickens present a compromised enzymatic antioxidant defense system with lower GSH‐PX activity (Dos Santos et al. 2012). However, there is no information available on how different combinations of Se sources, organic and inorganic, and muscle pH affect pork meat quality.

We hypothesize that in pigs, selenium source affects meat quality characteristics in a different way depending on the pH of the meat 24 h after slaughter. The objectives of the present research were to study the effect of the source of selenium supplementation in pigs (organic as Se‐enriched yeast vs. mineral as selenium selenite in feed) on some meat quality characteristics and the different response of selenium source according to muscle pH. Meat quality characteristics were measured as conductivity, drip loss, TBARS, and color changes.

## Material and Methods

All the experimental procedures used in this study comply with the Spanish guidelines for the care and use of animals in research (BOE 2013) and were in accordance with the protocols approved by the University Complutense of Madrid.

### Animals, experimental diets, and sample collection

Thirty pigs (Topigs 20 × Top York Topigs international) were housed in an environmentally controlled, slatted‐floor facility (COPISO, Soria, Spain). Pigs were fed a commercial diet that fulfilled the minimum NRC requirements (NRC 2012). At an average, live weight of 61.2 ± 0.5 kg pigs were randomly selected and distributed into two groups. Each pig was housed in an individual box (4.5 m^2^) during the experimental period. Experimental diets were formulated to provide nutrient composition above NRC (2012) recommendation diets (Table 1) and were identical in composition except for the selenium source: sodium selenite (Na_2_SeO_3_) (SeS) or selenomethionine from a Se‐enriched yeast diet (Sacharomices cervisae, Sel‐Plex; Alltech, Spain; Commission Regulation (EC) Nº 1750/2006) (SeY). In all cases, Se was introduced in the vitamin–mineral mix to achieve a final concentration of 0.3 mg/kg (Table 1). Pigs were fed the experimental diets until the end of the experiment (65 days in total), with 120.4 ± 2.5 kg. Food and water were provided ad libitum during the duration of the study.

**Table 1:** Ingredients and chemical composition of the experimental diets

Ingredients	
Wheat (%)	30.5
Barley (%)	20.2
Biscuit promic (%)	15.0
Pea 22/11, %	15.0
Sunflower 32 (%)	8.00
Colza 38 (%)	5.00
Fat 3/5	3.40
Soya bean 47	1.00
Calcium carbonate	0.95
l‐lysine 50 (%)	0.32
Premix^a^ (%)	0.20
Salt (%)	0.14
Bicalcium phosphate (%)	0.12
Enzyme pig	0.10
Bactericide	0.05
Fungicide	0.03
l‐Threonine (%)	0.02
Major nutrients	
Dry matter (%)	90.1
Crude protein (%)	15.1
Fat (%)	5.90
Crude fiber (%)	5.30
Starch (%)	43.9
Lysine dig (%)	0.70
Met dig (%)	0.21
Met + Cis dig (%)	0.50
ED, kcal/kg	3,317

a Premix (per kg of finished diet): Vitamin A: 4000000 IU; Vitamin D_3_: 900000 IU; Vitamin E (all rac *α*‐tocoferyl‐acetate): 7500 mg; Vitamin B_1_: 250 mg; Vitamin B_2_: 750 mg; Vitamin B_12_: 6 mg; Vitamin B_6_: 500 mg; Nicotinic acid: 7500 mg; Calcium Pantothenate: 5000 mg; Vitamin K_3_: 250 mg; Choline chloride: 50000; Fe (ferrous carbonate): 35000 mg; Cu (pentahydrate sulfate): 7500 mg; Co (hydrate carbonate): 25 mg; Zn (oxide): 50000 mg; Mn (oxide): 20000 mg; I (potassium iodure): 250 mg; Se (sodium selenite): 150 mg; 3‐fitase EC 1,6,3,2: 325000 FTU; BHT E321: 1000 mg; citric acid E330: 3450 mg; sodium citrate E331: 100 mg.

For organic selenium, supplementation reduced to 0. Se in basal diet previous to premix addition was 0.14 mg/kg.

At the end of the experiment period, pigs were sent to a commercial slaughterhouse (Incarlopsa, Tarancón, Cuenca, Spain) and slaughtered after a fasting period of 24 h. Carcasses were chilled (4°C) and samples, approximately 15 cm in size, were taken from the *Longissimus thoracis* muscle. Before packing in modified atmosphere (60–70% CO_2_, 30–40% N_2_, and <0.5% CO) and approximately 24 h after slaughter, electric conductivity (EC), and pH were measured by means of a LFStar conductivity meter (Mattahäus Ingenieurbüro, Klausa, DE) and a portable pH meter pH*K21 (NWK Binar, Puergen, DE), respectively.

### Laboratory analysis

#### Drip loss in muscle samples

Drip loss was estimated by the suspension method (Honikel et al. 1986). For the determination of weight loss during storage, approximately 1 cm^3^ samples (weighing approximately 10 g) were taken from the *Longissimus thoracis* muscle. After cutting, samples were weighed, put inside of a mesh and a plastic bag that was closed, and placed under refrigerated conditions at 4°C. Samples were weighed again at 72 h of storage. The difference between final and initial weights was used to calculate the drip loss that was expressed as a percentage of the initial weight.

Another piece of muscle was used for tocopherol and TBARS quantification and color measurement. Hence, 2‐cm‐thick samples were placed on trays, overwrapped with an oxygen‐permeable polyvinyl chloride wrap, and kept at 4°C under florescent light (600 lx) for the following determinations.

#### Tocopherol quantification in muscle samples

The *α*‐tocopherol concentration in muscle samples was quantified by direct extraction as described by Rey et al. (2010). Thus, muscle samples were mixed with 0.054 mol/L dibasic sodium phosphate buffer adjusted to pH 7.0 with HCl and absolute ethanol. After mixing, the tocopherol was extracted with hexane by centrifugation. The upper layer was evaporated to dryness and dissolved in ethanol prior to analysis. Tocopherols were analyzed by reverse phase HPLC (HP 1100, equipped with a diode array detector; Agilent Technologies, Waldbronn, Germany) as described elsewhere (Rey et al. 2010). Identification and quantification were carried out using a standard curve (*R* ^2^ = 0.999) of the pure compound (Sigma, Alcobendas, Madrid). All samples were analyzed in duplicate. The *α*‐tocopherol concentration in muscle was assessed on days 1 and 7 of refrigerated storage at 4°C.

#### TBARS analysis of muscle samples

Oxidation was assessed on days 1 and 7 by the thiobarbituric acid method described by Salih et al. 1987. A total quantity of 27 mL of perchloric acid (3.83% v/v) were added to 5 g of meat and the mixture was homogenized with an Ultra‐Turrax homogenizer for 1 min. and filtered through filter paper. Aliquots were added to thiobarbituric acid (0.02 mol/L) (1:1) and heated in boiling water for 15 min. A standard curve was prepared with 1,1,3,3‐tetraethoxypropane in water. Absorbance was measured at 532 nm and the values were expressed as mg MDA/kg meat.

#### Instrumental color analysis

The same 2‐cm‐thick samples placed on trays and kept at 4°C were used for color measurement. Muscle color was evaluated on days 1 and 7 after slaughter by means of a Chroma Meter (CM 2002, Minolta, Camera, Osaka, Japan) previously calibrated against a white tile in accordance with the manufacturer's recommendations (CIE 1976). The average of five random readings was used to measure lightness (L*), redness (a*), and yellowness (b*).

#### Statistical analysis

The experimental unit for analysis of all data was the pig. Data were analyzed following a completely randomized design using the general linear model procedure contained in SAS (1999) (version 9; SAS Inst. Inc., Cary, NC).

To study differences in pH, EC, and drip loss, dietary treatment was considered the fix effect according to the following model:

$$Y_{t}=\mu +\alpha_{t}$$

where *Y*_t_ is the dietary treatment‐dependent variable, *μ* the overall mean, and *α*_t_ the dietary treatment effect.

To compare differences in oxidation rate, vitamin E concentration, color parameters, and pigments between groups during time of refrigerated storage, dietary treatment, and time were considered fixed effects according to the following model:

$$Y_{\mathrm{ta}}=\mu +\alpha_{t}+\beta_{a}+{\left(\alpha \beta \right)}_{\mathrm{ta}}$$

where *Y*_ta_ is the dietary treatment or time response‐dependent variable, *μ* the overall mean, *α*_t_ the dietary treatment effect, *β*_a_ the effect of time (at which samples were performed), and the corresponding interaction (*αβ*)_ta_.

Data were presented as the mean of each group and root mean square error (RMSE) together with significance levels (*P* value). Differences between means were considered statistically significant at *P *<* *0.05.

The relationships between storage time (dependent variable) and muscle TBARS as well as pH (dependent variable) and drip loss were also quantified by regression equations (Statgraphics Centurion XVI, v. 16.1). A Student's *t*‐test was used to compare slopes of the regression equations.

## Results and Discussion

### Drip loss, electric conductivity, and pH in muscle samples

The EC and drip loss of the muscle samples from pigs fed with the experimental diets are presented in Table 2. Neither EC nor drip loss were affected by the selenium source. EC has been considered a reliable predictor of drip loss in pork muscle when measured at 24 h postmortem (Lee et al. 2000). Previous studies on the effect of diverse selenium sources on these meat quality characteristics showed different results. Some authors found that the drip loss was lower and water‐holding capacity was higher in pigs fed with the organic selenium (Mahan et al. 1999; Zhan et al. 2007; Li et al. 2011; Lisiak et al. 2014). However, other authors found a lack of effect in pork (Castro‐Ríos and Narvaéz‐Solarte 2013), or turkey meat (Juniper et al. 2011). The mechanism by which antioxidants modify drip loss and water‐holding capacity has been attributed to its capacity to stabilize membrane integrity postmortem (Ashgar et al. 1991), whereas others have proposed that proteolysis and even protein oxidation are key in influencing the moisture retention capacity of meat (Lonergan and Lonergan 2005). In this sense, it has been reported that the mechanism by which Se forms act are different. Organic Se (in the form of Se‐enriched yeast) is taken up via methionine transporter mechanisms and can be incorporated into selenoenzymes or in place of methionine in general body proteins (Suzuki and Ogra 2002). The replacement of Met by Se‐Met does not significantly alter protein structure, but may influence the activity of enzymes if Se‐Met replaces Met in the vicinity of the active site (Schrauzer 2000).

**Table 2:** Effect of selenium source (organic, SeY vs. mineral, SeS) on electric conductivity (EC) and pH at 24 h after slaughter, *α*‐tocopherol (*μ*g/g) and TBARS (mg MDA/kg meat) on days 1 and 7 of refrigerated storage in muscle samples from pigs fed with the experimental diets

	SeY	SeS	RMSE	*P*
EC24	3.54	3.84	1.046	0.3107
pH24	5.53	5.77	0.075	0.1648
Drip loss (%)	7.13	5.50	1.613	0.4834

RMSE, Root of the mean square error.

**Figure 1:**
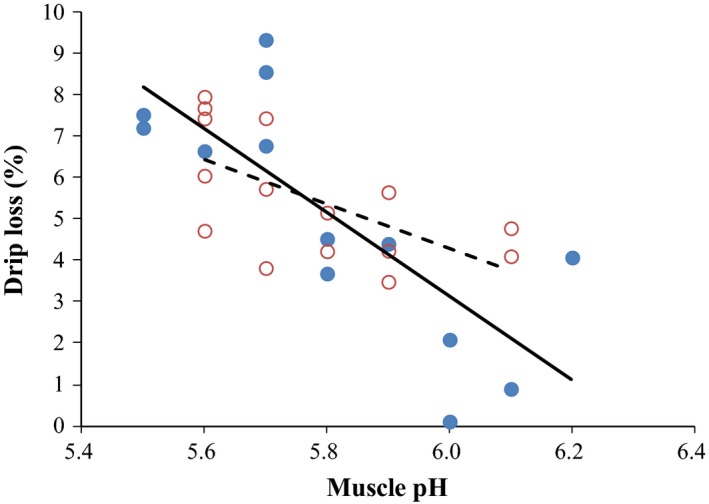
Drip loss (%) as affected by dietary treatment (organic Se — **●**; or inorganic Se ‐‐‐ **○**) and muscle pH (Organic Se = 80.72 (±14.79) −13.08 × pH (±2.56) a; *R* ^*2*^ = 0.72; RSD = 1.19; *P* = 0.0005; Inorganic Se = 36.01 (±10.95) −5.28 × pH (±0.74) b; *R* ^***2***^ = 0.37; RSD = 1.01; *P* = 0.015).

Drip loss depends on the shortening of sarcomeres which is regulated by the interaction of muscle temperature and rigor development (Fischer 2007) and on the interactions between water and protein, which is affected in turn by postmortem proteolysis. Consequently, the velocity and extent of the pH fall after slaughter are considered determinant for meat drip loss. Low pH (about 5.4–5.6) decreases muscle protein ability to bind to water as well as reduces negative electrostatic repulsion between filaments, diminishing the space between them and causing shrinkage of myofibrils (Den Hertog‐Meischke et al. 1997). To study with more detail the possible effect of muscle pH on the effect that dietary selenium source produce on meat quality characteristics, linear regression analysis was carried out. Muscle pH and drip loss were directly related by the linear equation: drip loss = 52.2 ± 9.2–8.11 pH ± 1.6 (*P* = 0.0001; *R* ^*2*^ = 0.50; RSD = 1.23). Hence, as the muscle pH increases, the drip loss proportion decreases in turn with previous studies (Fischer 2007). Moreover, in the regression study carried out, it can be observed a different response between dietary Se source and muscle pH (*R* ^*2*^ organic: 0.72, *P* = 0.0005; *R* ^*2*^ mineral: 0.37, *P* = 0.015) in which the slope (−13.08 ± 2.56 for the organic form vs. −5.25 ± 0.74 for the mineral form) was statistically different (Fig. 1). As the muscle pH increases, the drip loss decreases but this effect is more marked in meat from pigs that received organic Se in the diet (Fig. 1). There is no previous information on the possible interaction effects between pH and selenium source in the literature. A possible explanation is that calpain enzymes are particularly susceptible to inactivation by oxidation and muscle pH (Lonergan and Lonergan 2005) since they contain histidine and SH‐containing cysteine residues at their active sites. Calpain activation produces a rapid fragmentation of intermediate protein filaments in meat (such as desmin, which links myofibrils to the cell membrane), preventing shrinking of the overall muscle cell membrane (Lonergan and Lonergan 2005). Consequently, to maintain calpain activity reducing drip losses, a higher antioxidant balance and an enough high pH are needed. Hence, this study may point to a possible effect of pH on selenium biochemistry in muscle tissue. These results also suggest a different mechanism of Se on drip loss when compared to vitamin E which mainly act by protecting muscle membranes (Buckley et al. 1995) being consequently not muscle pH‐dependent.

### Tocopherol accumulation

Vitamin E concentration was measured taking into account the synergistic relationship that exists between selenium and vitamin E to protect against cellular damage caused by reactive oxygen species (Saito et al. 2003) and this synergism may enhance meat quality (Surai 2002). Concentrations of *α*‐tocopherol in muscle of pigs are presented in Tables 3 and 4. Organic or inorganic selenium supplementations to pigs resulted in similar concentrations of *α*‐tocopherol in muscle at day 1 and 7 of refrigerated storage. Although tocopherol concentrations were not different (*P* = 0.087), values in muscle samples from pigs fed with SeY seemed to be numerically higher with time of storage. There is not much available information on the possible effects of the selenium source on muscle vitamin E accumulation. It has been reported that absorption of vitamin E is impaired by severe Se deficiency, and Se alleviates such deficiency, permitting higher levels of vitamin E to be absorbed (Finkel and Holbrook 2000).

**Table 3:** Effect of selenium source (organic, SeY vs. mineral, SeS) and time on evolution of meat quality characteristics

	SeY	SeS	Initial	Final	RMSE^a^	RMSE^b^	Time	Se	Time*Se
TBARS, mg MDA/kg meat	0.16	0.19	0.02	0.33	0.002	0.002	0.0001	0.0130	0.0125
Muscle *α*‐tocopherol, *μ*g/g	3.03	2.49	3.42	2.11	1.610	0.945	0.0001	0.0845	0.3280
L* value	55.14	54.72	55.38	54.49	13.127	4.549	0.0774	0.9389	0.8475
a* value	6.77	5.55	7.02	5.30	2.116	0.827	0.0001	0.0027	0.7589
b* value	14.07	13.09	14.65	12.51	1.149	0.954	0.0001	0.0026	0.9320
Oxymyoglobin (OxyMb)	1.63	1.56	1.65	1.54	0.161	0.133	0.0001	0.0520	0.1926
Deoxymyoglobin (DeoxyMb)	1.05	1.05	1.04	1.05	0.948	0.942	0.2841	0.3282	0.3277
Metmyoglobin (MetMb)	0.87	0.87	0.84	0.90	1.145	1.123	0.0001	0.1335	0.1459

a RMSE: Root of the mean squares error from the main effect (selenium source).

b RMSE: Root of the mean squares error of the time and interactions.

**Table 4:** Effect of selenium source (organic, SeY vs. mineral, SeS) and pH on *α*‐tocopherol concentration, TBARS, and color (CIELAB L*, a*, b*, chroma, hue) of muscle samples from pigs fed with the experimental diets

	SeY	SeS	RMSE	Se	pH	Se × pH
Muscle *α*‐tocopherol, *μ*g/g						
Day 1	3.24	3.03	1.452	0.1284	0.2981	0.2070
Day 7	1.93	1.96	0.554	0.1093	0.8636	0.3615
Days 1–7	1.32	1.07	1.345	0.3191	0.3080	0.2805
TBARS refrigerated storage, mg MDA/kg meat						
Day 1	0.02	0.02	0.006	0.5375	0.5162	0.9651
Day 7	0.33	0.36	0.056	0.0087	0.1646	0.1471
L* value						
Day 1	54.82	54.18	2.057	0.9637	0.3396	0.2590
Day 7	52.26	51.52	3.143	0.8772	**0.0248**	0.1524
Days 1–7	2.55	2.66	2.923	0.8430	0.1261	0.5761
a* value						
Day 1	7.61	6.62	1.186	**0.0060**	0.8666	0.5080
Day 7	6.84	5.72	1.149	**0.0106**	**0.0129**	0.9815
Days 1–7	0.77	0.91	1.143	0.7303	**0.0233**	0.3872
b* value						
Day 1	15.10	14.19	0.901	**0.0121**	0.8056	0.9941
Day 7	13.05	11.49	1.118	**0.0307**	0.5546	0.0714
Days 1–7	2.06	2.70	1.366	0.9314	0.8411	0.1432
Chroma						
Day 1	16.94	15.68	1.252	**0.0850**	0.8042	0.8930
Day 7	14.95	13.09	0.916	**0.0006**	0.8996	0.4231
Days 1–7	1.99	2.59	1.295	0.9272	0.6685	0.3947
Hue						
Day 1	63.56	65.14	2.881	**0.0260**	0.7898	0.2741
Day 7	62.55	66.14	3.830	**0.0164**	**0.0202**	0.6141
Days 1–7	−1.01	1.00	3.215	0.2702	**0.0135**	0.8782

RMSE, Root of the mean square error.

L* = luminosity; a* = redness; b* = yellowness; Chroma = color saturation; Hue = tone.

Concerning the effect of storage time on *α*‐tocopherol concentration, it was found that as the storage time increased, the *α*‐tocopherol concentration was higher in muscle samples at day 1 when compared to day 7 (Table 3). These results were expected since vitamin E is the major lipid‐soluble antioxidant present in the cell membrane and plays an important role as a chain‐breaking lipid antioxidant and free radical scavenger in the membranes of cells and subcellular organs (Brigelius‐Flohé and Traber 1999). It is interesting to note the reduction in vitamin E along storage (*P* = 0.0001). Rey et al. (2010) also reported a 30% decrease in tocopherols concentration of dry‐cured ham slices during the first 7 days of storage. These authors also found that samples in which the level was initially higher had a more intense decrease. However, there is not more available information in the literature on the effects of storage time on *α*–tocopherol concentration in raw meat. In our study, no interactions were found between the time and selenium source of vitamin E levels. This lack of interaction effect was expected since vitamin E was not statistically affected by the selenium source.

Moreover, in our study, muscle pH did not affect vitamin E concentration (Table 4). A direct association between muscle vitamin E and lower drip loss has been described (Ashgar et al. 1991) and also a trend toward higher muscle pH at 24 h postmortem (5.7 vs. 5.5) (Lu et al. 2014); however, there is no further information on the possible interaction effects with the selenium source.

### TBARS in muscle samples

**Figure 2:**
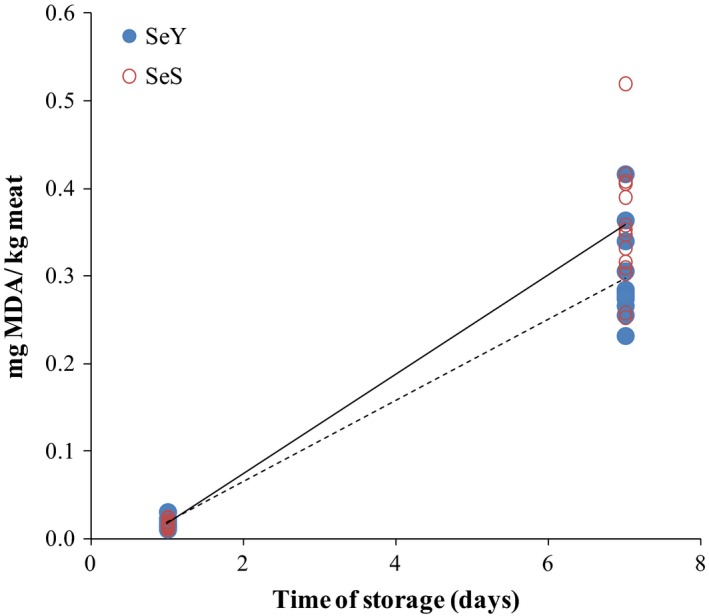
TBARS (mg MDA/kg meat) as affected by dietary treatment (organic Se — **●**; or inorganic Se ‐‐‐ **○**) and time (Organic Se = 0.027 (±0.012) +0.046 × days (±0.002) b; *R* ^*2*^ = 0.94; RSD = 0.02; *P* = 0.0001; Inorganic Se = 0.039 (±0.015) +0.057 × days (±0.003) a; *R* ^***2***^ = 0.93; RSD = 0.03; *P* = 0.0001).

To evaluate the oxidative status of pig meat according to the source of selenium supplementation, TBARS were measured in muscle (Tables 3 and 4). The group supplemented with SeY had lower malondialdehyde (MDA) concentration in muscle samples than the SeS group (*P* = 0.013) with the storage time (Table 3). The higher stability of muscle samples against lipid oxidation by SeY in pork has been reported previously (Zhan et al. 2007; Li et al. 2011). Similar results have been found in broiler (Chen et al. 2014) and turkey meat (Mikulski et al. 2009). Selenomethionine, the main form of organic Se present in Se‐enriched yeast, is an efficient scavenger of strong oxidant peroxynitrite, which is a product of nitric oxide and superoxide and capable of oxidizing a high variety of biomolecules (Padmaja et al. 1996). Moreover, the oxidative status of the samples was related to the storage time; as the time increased, the MDA concentration was higher in muscle samples from the SeS group when compared to that from the SeY group (interaction time x source; *P* = 0.0125) (Table 3, Fig. 2). Hence, the positive slope of the regression equation was lower for the SeY‐enriched pigs than for the SeS enriched. This result agrees with those that report the higher antioxidant activity of the organic Se and its more effective effects in delaying postmortem oxidation reactions (Mahan et al. 2014).

Moreover, TBARS numbers were not statistically affected by muscle pH (*P* = 0.16) and no interaction effect between selenium source and muscle pH was found. However, muscle samples with low pH had numerically higher TBARS than those with high pH. Juncher et al. (2001) reported that pork chops from groups with the lowest pH had higher TBARS and were less stable to oxidation compared to those with higher pH. It has been reported that the pH reduction could accelerate lipid oxidation due to the enhanced autoxidation of hemoglobin at reduced pH (Tsuruga et al. 1998). Consequently, TBARS development as affected by pH could also be studied by looking at its effect on color and pigment stability.

### Muscle color changes

Color changes, measured as L* (lightness), a* (red color), b* (yellow‐green color), chroma (saturation or color intensity), and hue (color tone) after 1 and 7 days of refrigerated storage, are presented in Table 3. The SeY group had higher a* values with storage time (*P* = 0.0027) than the SeS‐supplemented groups. The selenium source also affected the b* values similarly. Hence, pigs supplemented with Se‐enriched yeast diets had higher b* values than those from the SeS group with the storage time (*P* = 0.0026). These results are also observed in Table 4 where the Se and pH effect was evaluated. Chroma was also higher (*P* = 0.0006) on day 7 and hue lower on day 1 (*P* = 0.026) and 7 (*P* = 0.016) in SeY group when compared to SeS group (Table 4). The results of this study are also consistent with those observed for the TBARS numbers. The color stabilization effect of different dietary selenium sources has been reported by others (Bobcek et al. 2004; Zhan et al. 2007). Moreover, it has been reported that the inorganic form may have more prooxidant properties than the organic form (Spallholz 1994). Mahan et al. (1999) reported that pigs fed with inorganic Se had paler muscle tissue that increased linearly as dietary Se level increased, but not when pigs were fed organic Se. However, other authors have not found any effect of selenium on meat color (Lisiak et al. 2014). Juniper et al. (2011) suggested that once the Se content of tissue exceeds the requirements of antioxidant enzymes, further increases in tissue Se do not result in any noticeable improvement in meat quality. Furthermore, organic forms such as Se‐enriched yeast, are taken up via methionine transporter mechanisms and can be incorporated into selenoenzymes or in place of methionine into general body proteins (Suzuki and Ogra 2002), in a different way to the inorganic form (Surai 2006). Consequently, 0.3 mg/kg of the organic Se used in this study resulted in higher intensity of the color and redness than the inorganic form.

Moreover, muscle pH also influenced L* and a* values (Table 4). Hence, samples with low muscle pH had higher L* and lower a* values than those with high pH at day 7 of storage (*P* = 0.025 and *P* = 0.013, respectively). Losses in a* value (red color) with time were also higher in low pH muscle samples (*P* = 0.023). Color parameters were also not affected by Se and pH interaction as was observed for MDA content. The oxidation of oxymyoglobin to metmyoglobin which is accompanied by a change in the muscle redness is usually related to the muscle pH (James et al. 2002; Underland et al. 2004). This study provides novel information concerning the effect of Se and muscle pH. Zhan et al. (2007) reported that Se may slightly increase the pH value but no further research is available in the literature.

In conclusion, dietary organic selenium (SeY 0.3 mg/kg) improved the lipid stability of meat by reducing TBARS concentration with time when compared to the use of the inorganic sodium selenite. Dietary organic selenium also resulted in higher a*, b* values, and color intensity in pig meat. Moreover, regression equations show that there is a different response between dietary Se source and muscle pH; hence, dietary organic Se results in a more marked drip loss decrease as the muscle pH increases. This study may point to a possible effect of pH on selenium biochemistry in muscle tissue. Further research is needed to understand the specific effect of pH on the chemistry and metabolism of selenium.

## Conflict of Interest

This research has not conflict of interest.
